# Miniband and Gap Evolution in Gauss Chains

**DOI:** 10.3390/ma17184488

**Published:** 2024-09-12

**Authors:** D. S. Citrin

**Affiliations:** 1 School of Electrical and Computer Engineering, Georgia Institute of Technology, Atlanta, GA 30332-0250, USA; david.citrin@ece.gatech.edu; 2 Georgia Tech-CNRS IRL2958, Georgia Tech-Europe, 2 Rue Marconi, 57070 Metz, France

**Keywords:** quasiperiodic lattice, real-space renormalization group, Gauss chains, electronic structure

## Abstract

The Gauss chain is a one-dimensional quasiperiodic lattice with sites at $z_{j}=j^{n}d$, where j∈{0, 1, 2, …, N−1}, n∈{2, 3, 4, …}, and *d* is the underlying lattice constant. We numerically study the formation of a hierarchy of minibands and gaps as *N* increases using a Kronig–Penney model. Increasing *n* empirically results in a more fragmented miniband and gap structure due to the rapid increase in the number of minibands and gaps as *n* increases, in agreement with previous studies. We show that the Gauss chain $z_{j}=j^{2}d$ and a specific generalized Gauss chain, $z_{j}=\left(j^{2}\pm \frac{1}{2}j\right)d$, are treatable by a real-space renormalization group approach. These appear to be the only Gauss chains treatable by this approach, suggesting a hidden symmetry for the quadratic cases.

## 1. Introduction

One-dimensional quasiperiodic chains have attracted intense interest [1,2,3,4] spurred by the discovery of a quasicrystal by Shechtman et al. [5]. Quasiperiodic systems occupy an intermediate space between periodic and random systems, and they have attracted intense scientific interest for the light they shed on localized and delocalized states in solids. In addition, quasicrystals have begun to attract interest for applications [6,7]. They typically have low electrical and thermal conductivity, which makes them of interest for thermal insulation applications [8]. Their associations with other applications are attributable to their reduced adhesion and tribology properties [9].

We have been studying a novel class of quasiperiodic systems, namely Gauss chains (GCs). These are one-dimensional quasiperiodic lattices with sites at $z_{j}=j^{n}d$, where n∈{2, 3, 4, …} represents the order; j∈{0, 1, 2, …, N−1}, *N* represents the number of sites in the GC; and *d* represents the underlying lattice constant. GCs and similar structures have also been proposed, as seen in Refs. [10,11]. The appellation Gauss chain is due to the importance of Gauss sums [12] in the theory [13,14,15]. We have worked extensively on order n=2 GCs, viz. the quadratic GC, where we have shown that the structure factor is singular continuous in the N→∞ limit and also that the spectrum of delocalized electronic states in the GC exhibits a hierarchy of minibands and gaps [13,14,15]. GCs have interesting relationships with number–theoretic quantities [13,14] and thus are of mathematical interest. In addition, the identification of localized, critical, and extended states has been a focus of our work, as this classification impacts the transport properties of these structures. For example, extended states contribute to the conductivity in the large-*N* limit, but not delocalized states. We have also studied plasmonic GCs, suggesting possible applications for nanoscale filters [16].

Related structures that we have studied are (N+1)-site generalized quadratic GCs with $z_{j}=j^{2}d\pm jd^{\prime }$, with $j\in \{0,\;1,\;2,\;\ldots ,\;\frac{N}{2}-2,\;\frac{N}{2}-1,\;\frac{N}{2}\}$, focusing on the fundamental difference between commensurate and incommensurate generalized quadratic GCs, depending on whether *d* and $d^{\prime }$ are rationally related or not [14]. Commensurate generalized quadratic Gauss chains share many characteristics with quadratic GCs, whereas incommensurate generalized quadratic GCs do not. GCs and generalized quadratic GCs are of interest as they are a new class of quasiperiodic chains, but they are also of interest because of the role played by number–theoretic functions in their treatment.

Many of the remarkable properties of GCs follow from a study of their transfer matrix $T_{N}$ for an *N*-particle chain. Between the Gauss sites at $z_{j}$, a particle’s motion is unconstrained and can be characterized by its wavevector *k* in such regions, related to its dimensionless energy $\bar{E}$ (see below) by $kd=\sqrt{\bar{E}}$. The transfer matrix $T_{N}\left(k\right)$ depends on *k* and possesses a hidden *k*-dependent translational invariance [14]. That is, for $kd=k_{r,s}d=\frac{r}{s}\pi$ with *r* and *s* coprime integers, $T_{ms}\left(k_{r,s}\right)={\left[T_{s}\left(k_{r,s}\right)\right]}^{m}$, where *m* is a positive integer. Thus, for a given $k_{r,s}$, the GC in effect has a periodicity similar to that of a periodic crystal; however, the GC exhibits different periodicities depending on *s*. This hidden translational invariance is shown explicitly below.

**Figure 1:**
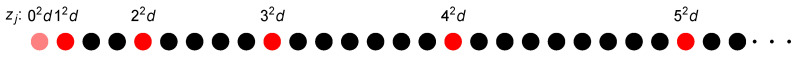
Schematic diagram of a GC with n=2. An *N*-site GC has Gauss sites (red) at $z_{0}$ to $z_{N-1}$. The site for j=0, shown in pink, has half the form factor as the other Gauss sites. In the present work, we employ a continuum Kronig–Penney model with Dirac δ-function potentials on the Gauss sites and zero potential on the sites between them. The atomic form factor for the red sites is unity.

Figure 1 shows a schematic diagram of an *N*-site GC with n=2. We have fully and analytically quantified the delocalized states in the N→∞ limit in a Kronig–Penney model for this GC [14,15]. While we have presented some numerical results for miniband and gap formation for finite GC length as *N* increases, little insight was provided at the time that work was published [14]. It has also been observed that the hierarchy of minibands and gaps becomes more fragmented as *n* increases [17]. Our aim here is to explore miniband and gap formation with increasing *N*. This is analogous to how bands form in finite but otherwise periodic crystals as the crystal size increases. Understanding how minibands and gaps form in GCs may, in the future, enable a rigorous classification of the minibands and allow for a better understanding of the formation of extended states as N→∞. Here, we carry out numerical work showing that the strongly fragmented nature is connected with the rapid increase in the number of minibands and gaps with increasing *n*. The behaviors for various values of *n* are also investigated.

We also formulate a bottom-up real-space renormalization-group (RSRG) approach applicable to a case where n=2, complementary to that used in Ref. [18]. There, however, we applied a top-down approach to analyze the electronic structure of long yet finite-length GCs (in a nearest-neighbor tight-binding model). By “bottom-up”, we mean that the *N*-site GC half-trace $x_{N}=\frac{1}{2}\mathrm{Tr}T_{N}$ of the transfer matrix $T_{N}$ is built up by an inflation rule starting with N=1; by a top-down approach, we ultimately reduce the *N*-site transfer matrix to a one-site transfer matrix with renormalized parameters by means of a deflation rule. Studying $x_{N}\left(k_{r,s}\right)$ is key to understanding the localization properties of the states at $k_{r,s}$. For $|x_{N}\left(k_{r,s}\right)|\;>1$, the state is localized; for $|x_{N}\left(k_{r,s}\right)|\;<1$, it is extended; and for $|x_{N}\left(k_{r,s}\right)|\;=1$, it is critical. This is discussed further below. Here, we devise an iterative relation for the half-trace $x_{N}$ of the transfer matrix $T_{N}$, as *N* is incremented, and use this to characterize the minibands and gaps observed. We do not use the RSRG approach for the numerics; however, the approach may be connected to why the case n=2 and the generalized quadratic GC we study might be special in leading to a clear hierarchy of minibands and gaps. This is apparently the only case for which our RSRG approach works, perhaps reflecting the special symmetry of this specific case. The RSRG approach may also provide insight into the self-similar properties of the GC and its electronic properties.

The remainder of this paper is organized as follows: In Section 2, we review the theoretical approach that leads to a transfer matrix $T_{N}$ for the GC. Section 3 outlines how we can obtain an iterative relation for the half-trace $x_{N}$ of $T_{N}$ for the GC of length *N*. Section 4 presents numerical results, while Section 5 gives our conclusions.

## 2. Methods

### 2.1. Setting Up the Problem

We employ a Kronig–Penney model as the basis of a transfer matrix approach [19]. The effective-mass Hamiltonian for an electron in an *N*-site GC is

(1) $$\begin{array}{ccc} H & = & -\frac{\hbar^{2}}{2m^{*}}\frac{\partial^{2}}{\partial z^{2}}+\frac{\hbar^{2}}{2m^{*}}\frac{\lambda }{d}f\left(z\right), \end{array}$$

(2) $$\begin{array}{ccc} f(z) & = & \sum_{j=0}^{N-1}\delta \left(z-z_{j}\right)\left(1-\frac{1}{2}\delta_{j,0}\right) \end{array}$$

where $m^{*}$ is the effective mass, and λ is the dimensionless strength of the potential at the Gauss sites. Site j=0 has half the weight of the others so that the Fourier transform of f(z) is proportional to a Jacobi theta function [13] for n=2. In any case, this has little effect on the results. For $z<z_{0}$ and $z>z_{N}$, we employ free-particle boundary conditions. We dispense with excess parameters by defining the normalized Hamiltonian $\bar{H}=\frac{2m^{*}}{\hbar^{2}}H$. We then solve the Schödinger equation $\bar{H}\psi =\bar{E}\psi$, where $\bar{E}$ is an eigenvalue (dimensionless) of $\bar{H}$ and ψ is the corresponding eigenfunction.

### 2.2. Transfer Matrix

The transfer matrix $T_{N}$ for the *N*-site GC relates the left- and right-going travelling-wave components of the free-particle state in $z>z_{N}$ to those in $z<z_{0}$. In this study, we are mainly interested in delocalized states. We define $kd=\sqrt{\bar{E}}$; *k* to be the wavevector of an eigenfunction of $\bar{H}$ between successive Gauss sites where the electron behaves as a free particle. Without loss of generality, we take d=1 and associate $k=\sqrt{\bar{E}}$ with a dimensionless wavevector. We use the transfer matrix approach discussed in Ref. [20], also called the *M*-matrix in Ref. [19]. Consider the wavefunction near site $z_{j}$ for some j∈{0, 1, 2, …, N−1}. For $z_{j-1}<z<z_{j}$, the wavefunction is of the form $\psi \left(z\right)=A_{j}e^{-ikz}+B_{j}e^{ikz}$, where $A_{j}$ and $B_{j}$ unknown coefficients. Based on the continuity of ψ at $z_{j}$ and by integrating the Schrödinger equation from $z_{j}-\epsilon$ to $z_{j}+\epsilon$ for ϵ→0, we have

(3a) $$\begin{array}{ccc} A_{j+1}e^{-ikz_{j}}+B_{j+1}e^{ikz_{j}} & = & A_{j}e^{-ikz_{j}}+B_{j}e^{ikz_{j}}, \end{array}$$

(3b) $$\begin{array}{ccc} A_{j+1}e^{-ikz_{j}}-B_{j+1}e^{ikz_{j}} & = & \left(-\frac{\lambda }{ikd}+1\right)A_{j}e^{-ikz_{j}}\\ & & +\;\left(-\frac{\lambda }{ikd}-1\right)B_{j}e^{ikz_{j}}, \end{array}$$

which can be rearranged in matrix form to obtain

(4) $$\left[\begin{array}{c} A_{j+1}e^{-ikz_{j}}\\ B_{j+1}e^{ikz_{j}} \end{array}\right]=M\left[\begin{array}{c} A_{j}e^{-ikz_{j}}\\ B_{j}e^{ikz_{j}} \end{array}\right]$$

with

(5) $$M=\left[\begin{array}{cc} 1+\gamma & \gamma \\ -\gamma & 1-\gamma \end{array}\right],\;M_{0}=\left[\begin{array}{cc} 1+\gamma /2 & \gamma /2\\ -\gamma /2 & 1-\gamma /2 \end{array}\right].$$

*M* is the transfer matrix across Gauss sites other than j=0, while $M_{0}$ is the transfer matrix across sites j=0 and $\gamma =i\frac{\lambda }{2kd}$.

The transfer matrix for free propagation between successive Gauss sites $z_{j}$ and $z_{j+1}$ is

(6) $$Q_{j}=\left[\begin{array}{cc} \exp[-ik\left(z_{j+1}-z_{j}\right)] & 0\\ 0 & \exp[ik\left(z_{j+1}-z_{j}\right)] \end{array}\right].$$

Finally, the transfer matrix of the *N*-site GC is as we showed in Ref. [13]

(7) $$T_{N}=Q_{N-1}MQ_{N-2}M\ldots MQ_{1}MQ_{0}M_{0}.$$

Notice that $Q_{j+s}\left(k_{r,s}\right)=Q_{j}\left(k_{r,s}\right)$, from which the hidden *k*-dependent translational invariance $T_{ms}\left(k_{r,s}\right)={\left[T_{s}\left(k_{r,s}\right)\right]}^{m}$ follows for *m*, a positive integer.

Since *M*, $M_{0}$, and $Q_{j}$ are unimodular, so is $T_{N}$. This means the eigenvalues $\xi_{\pm }$ of $T_{N}$ (not of $\bar{H}$) satisfy $\xi_{+}\xi_{-}=1$. Since the trace of a matrix is invariant under similarity transformations, the half-trace $x_{N}$ of $T_{N}$ satisfies $2x_{N}=\mathrm{Tr}T_{N}=\xi_{+}+\xi_{-}$ [21]. Delocalized states (extended and critical) then correspond to energies $\bar{E}$ with $k=\sqrt{\bar{E}}$, for which $|x_{N}|\;\leq 1$. The *k*-dependence of $x_{N}$ is understood in these and similar expressions even if not explicitly written. It would be useful to plot quantities related to $\left[|x_{N}|\;\leq \;1\right]$, where [P] is the Iverson bracket, i.e., [P]=1 if proposition *P* is true, and [P]=0 if *P* is false.

A few comments on the symmetry properties of $T_{N}$ are in order. It is useful to make an approximation whereby we neglect the *k*-dependence of *M* and $M_{0}$ but retain the *k*-dependence of $Q_{j}$. This not only allows us to facilitate numerical computation but also enables us to highlight the basic characteristics of the miniband and gap formation, which might be obscured by including this *k*-dependence. Consider k∈[mπ, (m+1)π), where m≫1 for n=2. With regard to the approximation just stated, we can approximate $\gamma \approx i\frac{\lambda }{2m\pi }$ in that interval, neglecting the *k*-dependence of γ and, hence, that of *M* and $M_{0}$. Note that the approximation improves as *m* increases. We label the transfer matrix under this approximation as ${\tilde{T}}_{N}$ and further define ${\tilde{x}}_{N}=\frac{1}{2}\mathrm{Tr}{\tilde{T}}_{N}$. $\left[|{\tilde{x}}_{N}|\;\leq \;1\right]$ is periodic in *k* with period π for n=2, and thus, we will provide plots for k∈[0, π). Consequently, we set γ to be constant in the sequel, choosing $\gamma =\frac{i}{2}$. Note that making the replacements γ→−γ and k→−k in the modified transfer matrix ${\tilde{T}}_{N}$ takes ${\tilde{T}}_{N}$ to ${\tilde{T}}_{N}^{-1}$, thus changing the sign of γ, preserving the solutions of $|{\tilde{x}}_{N}|\;\leq \;1$. This approximation is not terribly severe, and it is easily relaxed for numerical computations. We employ it, however, because it emphasizes the structure of the miniband and gap formation as *N* increases.

In the next section, we discuss how to compute $x_{N}$ or ${\tilde{x}}_{N}$ without explicitly calculating the matrix product in Equation (7) for the n=2 case.

### 2.3. Real-Space Renormalization Group and Recurrence Relation for $x_{N}$

In this section, we will use a self-similar property of the GC for n=2 to obtain an iterative relation for $x_{N}$ or ${\tilde{x}}_{N}$, thus precluding the necessity of explicitly computing the matrix product in Equation (7). We do not use the RSRG for the numerical computations; however, the fact that the approach works only for n=2 is attributable to the self-similarity that is also only exhibited for this case. We have not found a way to apply this approach to other values of *n*. A top-down version of the approach is discussed in Ref. [18]. Here, we want to start with N=1 and explore the delocalized-state spectrum as *N* is incremented in unit intervals.

To proceed, let us denote the (p, q)-th entries of *M* and $M_{0}$ as $m_{pq}$ and $m_{0,pq}$, respectively. We will later restore the values of the matrix elements given in Equation (5). Note that the following inflation rule can be used to generate the transfer matrix for an (N+1)-site GC from an *N*-site chain:

(8) $$\begin{array}{ccc} \;M & \to & Q_{0}MQ_{0}, \end{array}$$

(9) $$\begin{array}{ccc} M_{0} & \to & Q_{0}MQ_{0}M_{0}Q_{0}. \end{array}$$

For example, after one application of this rule to $T_{N}$ in Equation (7), we have

(10) $$\begin{array}{ccc} & & Q_{N-1}\left(Q_{0}MQ_{0}\right)Q_{N-2}\left(Q_{0}M\ldots MQ_{0}\right)Q_{1}\left(Q_{0}MQ_{0}\right)Q_{0}\left(Q_{0}MQ_{0}M_{0}Q_{0}\right)\\ & = & Q_{N-1}Q_{0}MQ_{N-1}M\ldots MQ_{2}MQ_{1}MQ_{0}M_{0}Q_{0}. \end{array}$$

We define the equivalence class of transfer matrices obtained by cyclic permutations of the constituent factors since two such transfer matrices have the same half-trace $x_{N}$. If matrices *A* and *B* are in the same equivalence class, we say *A* is equivalent to *B* and write A≡B. That is, inasmuch as we are concerned with the half-trace $x_{N+1}$ of the transfer matrix $T_{N+1}$, we will cyclically permute the final factor of $Q_{0}$ to the front of the product to give

(11) $$\begin{array}{ccc} T_{N+1} & = & Q_{N}Q_{0}MQ_{N-1}M\ldots MQ_{2}MQ_{1}MQ_{0}M_{0}\\ & & \equiv Q_{N-1}Q_{0}MQ_{N-1}M\ldots MQ_{2}MQ_{1}MQ_{0}M_{0}Q_{0}. \end{array}$$

We can abbreviate this inflation rule as follows:

(12) $$x_{N+1}\left[M,M_{0}\right]=x_{N}\left[Q_{0}MQ_{0},Q_{0}MQ_{0}M_{0}Q_{0}\right].$$

By computing $Q_{0}MQ_{0}$ and $Q_{0}MQ_{0}M_{0}Q_{0}$ in terms of the $m_{pq}$ and $m_{0,pq}$, the rules (8) and (9) are equivalent to making the following replacements in $x_{N}\left[M,M_{0}\right]$ to obtain $x_{N+1}\left[M,M_{0}\right]$:

(13) $$\begin{array}{ccc} \;m_{11} & \to & m_{11}e^{-i2kd}, \end{array}$$

(14) $$\begin{array}{ccc} \;m_{12} & \to & m_{12}, \end{array}$$

(15) $$\begin{array}{ccc} \;m_{21} & \to & m_{21}, \end{array}$$

(16) $$\begin{array}{ccc} \;m_{22} & \to & m_{22}e^{i2kd}, \end{array}$$

(17) $$\begin{array}{ccc} \;m_{0,11} & \to & \left(m_{11}m_{0,11}e^{-i2kd}+m_{12}m_{0,21}\right)e^{-ikd}, \end{array}$$

(18) $$\begin{array}{ccc} m_{0,12} & \to & \left(m_{11}m_{0,12}e^{-i2kd}+m_{12}m_{0,22}\right)e^{ikd}, \end{array}$$

(19) $$\begin{array}{ccc} m_{0,21} & \to & \left(m_{22}m_{0,21}e^{i2kd}+m_{21}m_{0,11}\right)e^{-ikd}, \end{array}$$

(20) $$\begin{array}{ccc} \;m_{0,22} & \to & \left(m_{22}m_{0,22}e^{i2kd}+m_{21}m_{0,12}\right)e^{ikd}. \end{array}$$

Thus, in terms of the parameters, we can write down the iterative relation

(21) $$\begin{array}{c} \;x_{N+1}\left(m_{11},m_{12},m_{21},m_{22},m_{0,11},m_{0,12},m_{0,21},m_{0,22}\right)\\ \;=x_{N}\left(m_{11}e^{-i2kd},m_{12},m_{21},m_{22}e^{i2kd},\right.\\ \;\left.\left(m_{11}m_{0,11}e^{-i2kd}+m_{12}m_{0,21}\right)e^{-ikd},\left(m_{11}m_{0,12}e^{-i2kd}+m_{12}m_{0,22}\right)e^{ikd},\right.\\ \;\left.\left(m_{22}m_{0,21}e^{i2kd}+m_{21}m_{0,11}\right)e^{-ikd},\left(m_{22}m_{0,22}e^{i2kd}+m_{21}m_{0,12}\right)e^{ikd}\right) \end{array}$$

with the initial condition

(22) $$x_{1}\left(m_{11},m_{12},m_{21},m_{22},m_{0,11},m_{0,12},m_{0,21},m_{0,22}\right)=\frac{1}{2}\left(m_{0,11}e^{-ikd}+m_{0,22}e^{ikd}\right).$$

The expressions above also hold for ${\tilde{x}}_{N}$. This bottom-up RSRG approach obviates the need to explicitly compute transfer matrices. To conclude this section, the iterative approach just discussed works for n=2. We have not seen how to generalize the technique to n>2.

## 3. Results

In this section, we numerically study the delocalized-state spectrum for the GC as a function of *N* for n=2 and 3. In addition, below, we also consider the generalized quadratic GC mentioned above. In all cases henceforth, without loss of generality, we remind the reader that *d* is set to be equal to one.

### 3.1. GC with n=2

**Figure 2:**
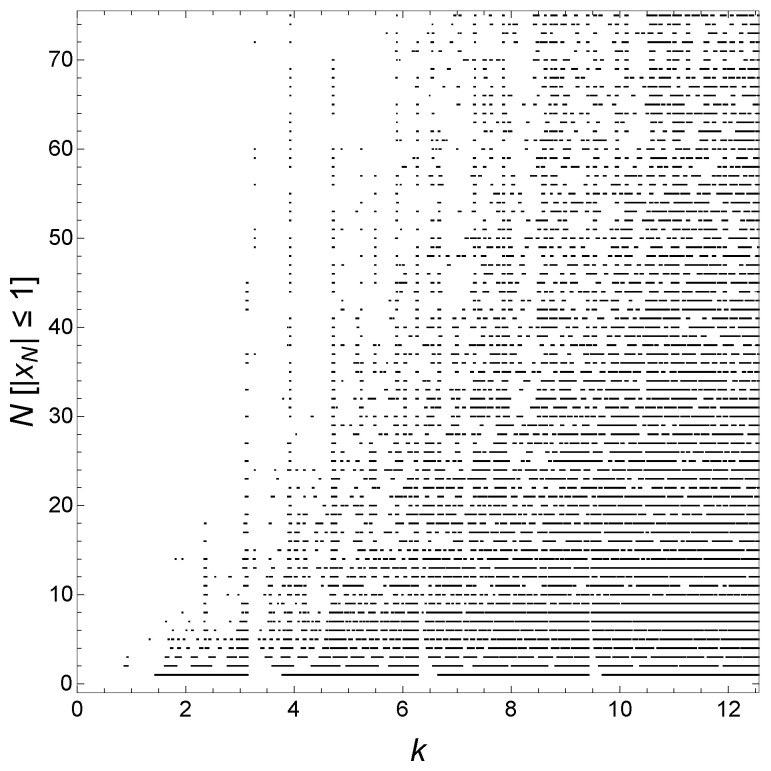
$N[|x_{N}|\leq 1]$ as functions of *k* for various *N*. This gives the wavevectors $k=\sqrt{\bar{E}}$ of delocalized states for various *N* here with n=2, d=1, and λ=5. [P] is an Iverson bracket with [P]=1 if proposition *P* is true and [P]=0 if it is false.

Figure 2 plots the values of $k=\sqrt{\bar{E}}$, for which the state is delocalized for n=2, with λ=5 illustrating the development of the hierarchy of minibands and gaps as *N* increases. Specifically, what is plotted is $N[|x_{N}|\leq 1]$, i.e., the product of *N* and the Iverson bracket $\left[|x_{N}|\leq 1\right]$, as a function of *k* for various *N*. The inclusion of the factor *N* visually separates the results for the minibands and gaps for various *N*. In other words, for a given value of *k*, if $|x_{N}\left(k\right)|\leq 1$, we plot a black point at (k, N) in the plane. We have chosen the onsite potential on Gauss sites to be repulsive, but the results are similar for attractive potentials. States tend to become localized with increasing *N*, showing more sparse behavior as *N* increases, i.e., as one moves upward in the plot, though some states remain delocalized in the limit N→∞, as expected based on our work in Refs. [14,15]. Also note the vague appearance of approximate periodicity in *k* with period π, most evident for low values of *N*. For example, note the pronounced gaps near integer multiples of π. The *k*-dependence of γ included here is responsible for the lack of strict periodicity. If the *k*-dependence of γ is neglected, this diagram would be strictly periodic in *k* with period π; see below. As *N* increases, a number of features persist indicating delocalized states, though this will be made clearer below. In particular, states $k_{r,1}$ are trivially delocalized because we can construct standing waves for such *k* with nodes at the Gauss sites.

**Figure 3:**
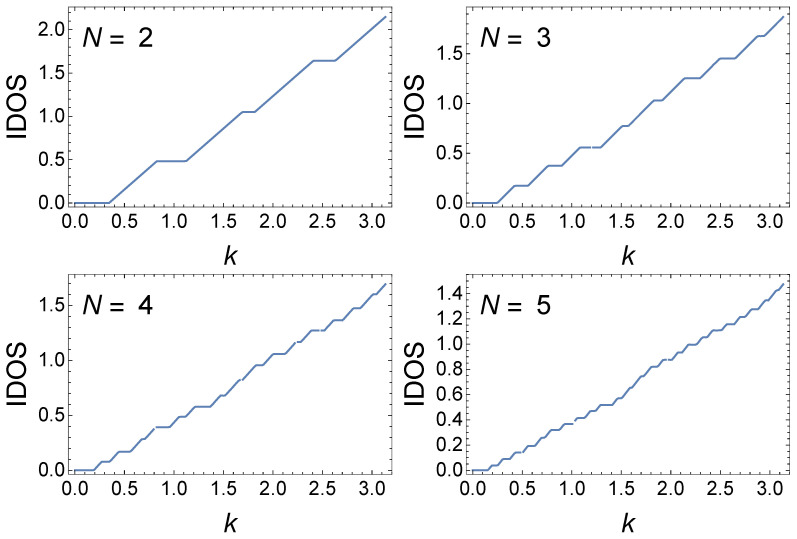
IDOS(k) for an n=2 GC for various *N* with $\gamma =\frac{i}{2}$.

**Figure 4:**
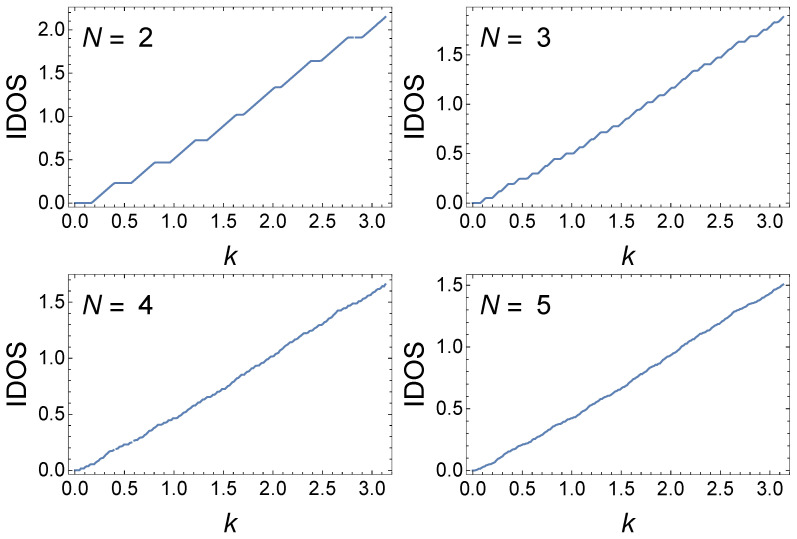
IDOS(k) for an n=3 GC for various *N* with $\gamma =\frac{i}{2}$.

**Figure 5:**
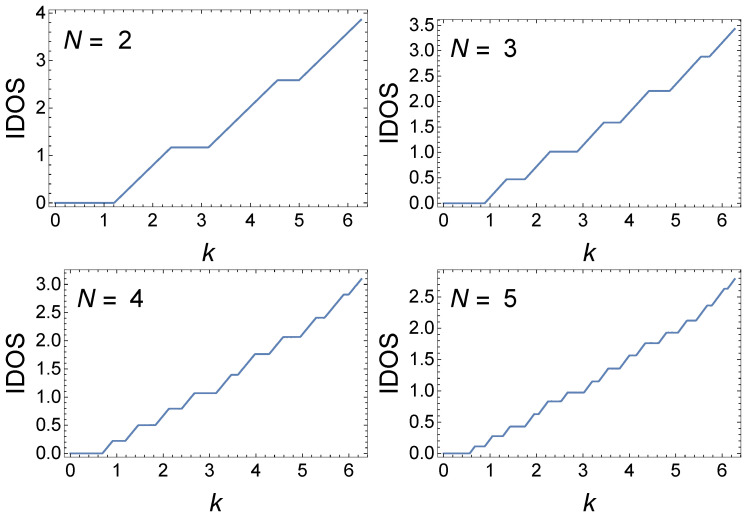
IDOS(k) for an GC with sites at $z_{j}=\left(j^{2}\pm \frac{1}{2}j\right)d$ for various *N* with $\gamma =\frac{i}{2}$, computed with $\gamma =\frac{i}{2}$. This is a GC with sites at $(j^{2}\pm \frac{1}{2}j)d$, but the sites are ordered from smallest $z_{j}$ to largest. The period is 2π.

**Figure 6:**
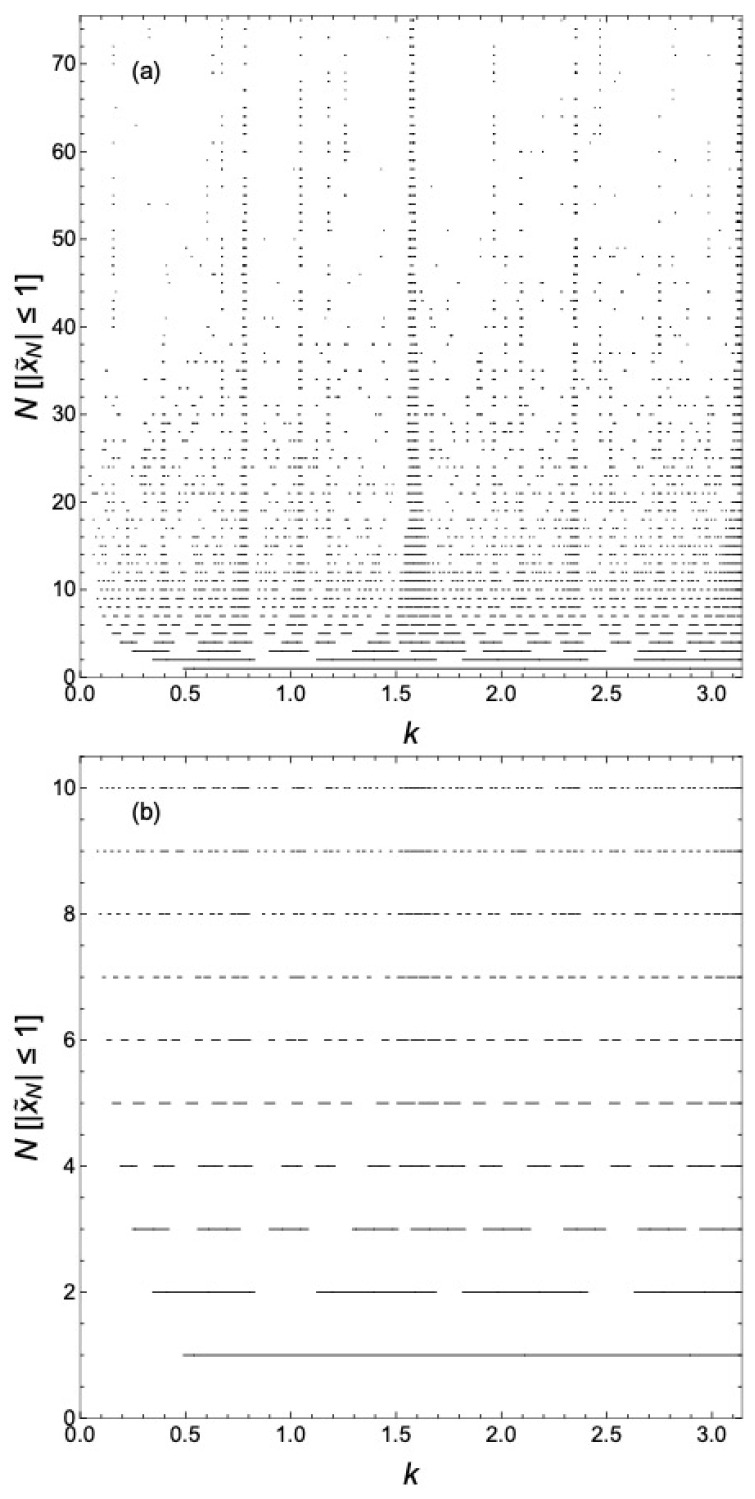
$N[|{\tilde{x}}_{N}|\leq 1]$ as functions of *k* for various *N*. This gives the wavevectors $k=\sqrt{\bar{E}}$ of delocalized states for various *N* here with n=2, d=1, and $\gamma =\frac{i}{2}$. [P] is an Iverson bracket with [P]=1 if proposition *P* is true and [P]=0 if it is false. (**a**) Global view; (**b**) data for N=1 to 10.

**Figure 7:**
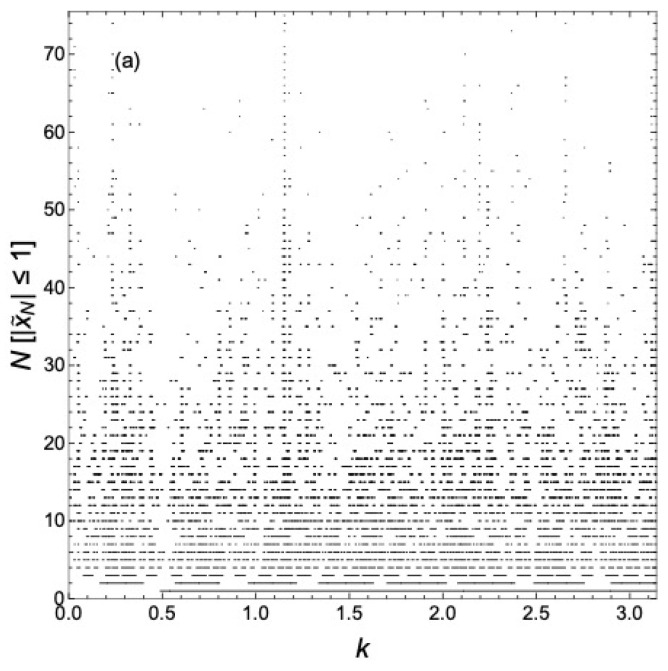

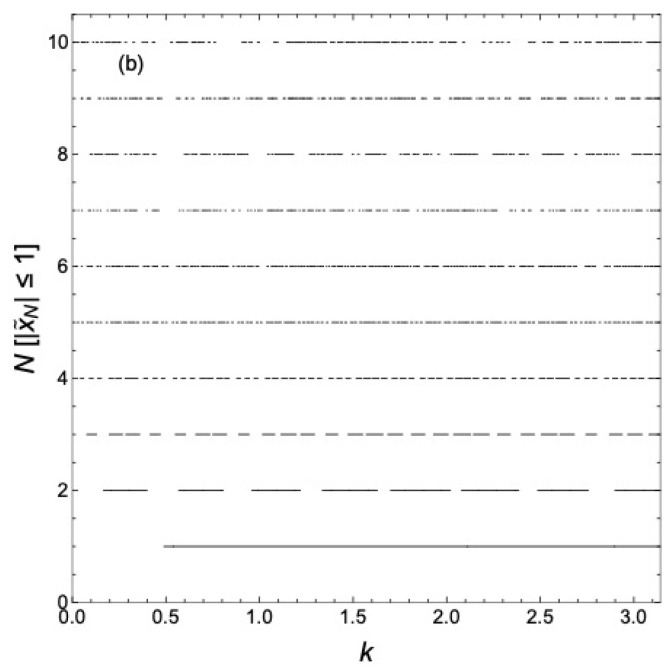
$N[|{\tilde{x}}_{N}|\leq 1]$ as functions of *k* for various *N*. This gives the wavevectors $k=\sqrt{\bar{E}}$ of delocalized states for various *N* here with n=3, d=1, and $\gamma =\frac{i}{2}$. [P] is an Iverson bracket with [P]=1 if proposition *P* is true and [P]=0 if it is false. (**a**) Global view; (**b**) data for N=1 to 10.

**Figure 8:**
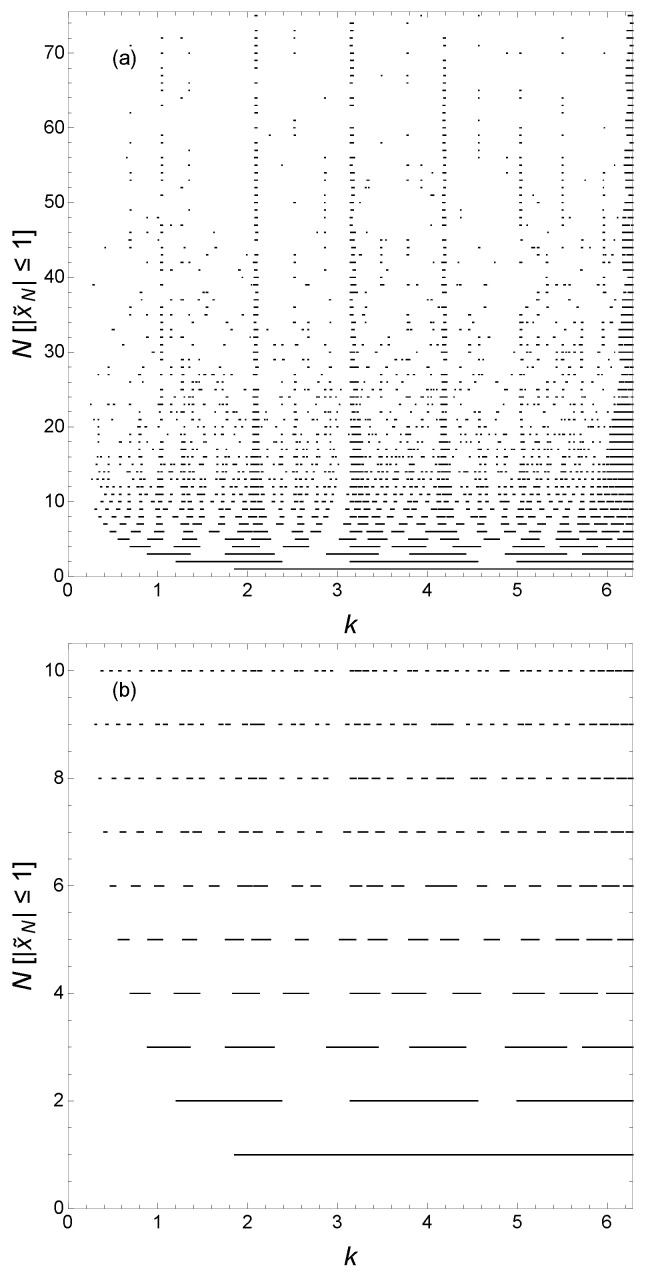
$N[|{\tilde{x}}_{N}|\leq 1]$ as functions of *k* for various *N*. This gives the wavevectors $k=\sqrt{\bar{E}}$ of delocalized states for various *N* here with sites at $z_{j}=\left(j^{2}\pm \frac{1}{2}j\right)d$, d=1, and $\gamma =\frac{i}{2}$. [P] is an Iverson bracket with [P]=1 if proposition *P* is true and [P]=0 if it is false. (**a**) Global view; (**b**) data for N=1 to 10.

In the ensuing calculations, we neglect the *k*-dependence of γ to emphasize the evolution of the hierarchy of minibands and minigaps with increasing *N*. The inclusion of the full *k*-dependence merely distorts the picture without changing its hierarchical nature. We then consider the *N*-dependence of the integrated density of (delocalized) states, $\mathrm{IDOS}(\bar{E})$, defined as

(23) $$\begin{array}{ccc} \;\mathrm{IDOS}(\bar{E}) & = & \int_{0}^{\bar{E}}d{\bar{E}}^{\prime }\varrho \left({\bar{E}}^{\prime }\right), \end{array}$$

(24) $$\begin{array}{ccc} \varrho (\bar{E}) & = & \sum_{k^{\prime }}\delta \left[\bar{E}-\bar{E}\left(k^{\prime }\right)\right] \end{array}$$

where $\varrho (\bar{E})$ is the density of delocalized states. Evaluating the integral in Equation (23) and converting IDOS to a function of *k* gives $\mathrm{IDOS}\left(k\right)=\sum_{k^{\prime }=0}^{k}\left[x_{N}\leq 1\right]$ or $\sum_{k^{\prime }=0}^{k}\left[{\tilde{x}}_{N}\leq 1\right]$ whether or not we retain the *k*-dependence of γ. In Figure 3, Figure 4, Figure 5, Figure 6, Figure 7 and Figure 8, we plot quantities neglecting the *k*-dependence of γ to bring out the generic features of the structure of the minibands and gaps. These features persist when the *k*-dependence is included but might be distorted and less clear. Figure 3 shows IDOS(k) for n=2 and N=2, 3, 4, and 5 for $\gamma =\frac{i}{2}$. IDOS(k) shows successive intervals of slope 1 and 0; the former correspond to minibands, and the latter to gaps. Apart from the lack of *k*-dependence in *M* and $M_{0}$, these plots contain the same information as in Figure 2, albeit only for selected small values of *N*. Expanding to first order in γ, we see that ${\tilde{x}}_{N}\approx \cos N^{2}k-i\gamma N\sin N^{2}k$. From $\left[|x_{N}|\leq 1\right]$, this, in turn, implies that there are $N^{2}$ minibands and $N^{2}$ gaps. As *N* increases, the slopes and plateaux consequently increase in number with $N^{2}$ each associated with a miniband and gap, respectively. For n=2, we see a feature near $k=\frac{\pi }{2}$ that persists as *N* increases; such a feature, as we shall see, does not appear for n>2.

### 3.2. GC with n=3

The case of n=3 is shown in Figure 4 and shows similar if more fragmented behavior; one shows that there are $N^{3}$ slopes/plateaux. Because this number of minibands and mingaps increases rapidly with *N*, the miniband and gap structure rapidly become broken up with increasing *N*—with this happening far faster than in the case of n=2. This trend continues successively intensifying with increasing *n*.

### 3.3. Generalized GC with n=2

Next, we consider the generalized quadratic GC with $z_{j}=\left(j^{2}\pm \frac{1}{2}j\right)d$. Figure 5 shows IDOS(k) for this GC in k∈[0, 2π) since $x_{N}$, in this case, is periodic with period 2π. Indeed, we see an even less fragmented structure of minibands and gaps than for n=2. In this case, for each *N*, there are $\frac{1}{2}N\left(N+1\right)$ minibands and gaps. Further comparison of these cases is presented in the next subsection.

## 4. Discussion

It is easier to visualize the continuing evolution with *N* by plotting $N[|{\tilde{x}}_{N}|\leq 1]$ for various *N* in the same plot for each case considered above, as we will now show. That is, we neglect the *k*-dependence of γ to better bring out the structure of the minibands and gaps and better illustrate their evolution as *N* increases, in contrast to what is shown, say, in Figure 2. A global view of the delocalized states as a function of *k* over one period in *k* for various *N* is shown in Figure 6a for n=2. That is, what is plotted is $N[|{\tilde{x}}_{N}|\leq 1]$ as functions of *k* for various *N*. We see what is apparently a self-similar hierarchy of minibands and gaps developing as *N* increases. The number of minibands in one period in k∈[0,π) is observed to be $N^{2}$. A particularly pronounced miniband occurs near $k=\frac{\pi }{2}$, and this miniband persists as *N* increases. We have not, however, been able to classify the minibands and gaps as reviewed in Ref. [4] for Fibonacci chains. There is no known analogous gap-labeling theorem [22,23,24] for GCs.

Figure 7 shows $N[|{\tilde{x}}_{N}|\leq 1]$ as functions of *k* for various *N* for the case n=3, viz. $z_{j}=j^{3}d$, for which $z_{j+1}-z_{j}=3j\left(j+1\right)+1$, again with $\gamma =\frac{i}{2}$. The energy eigenvalues are periodic in *k* with period π; however, the term 3j(j+1) in $z_{j+1}-z_{j}=3j\left(j+1\right)+1$ also produces features on the scale of changes in *k* of $\frac{\pi }{6}$ as j(j+1) is even. In Figure 7a, we can see these underlying features giving an overall fragmented appearance to the hierarchy of minibands and gaps. The fragmentation of the delocalized states for cases n>2 has been documented in Ref. [17].

One can additionally consider GCs for which $z_{j}$ is not simply a power of *j*, but instead a polynomial with commensurate coefficients, i.e., a commensurate generalized quadratic GC. (For the incommensurate case, the electronic structure is not periodic in *k* and is highly fragmented [14].) Figure 8 shows $N[|{\tilde{x}}_{N}|\leq 1]$ as functions of *k* for various *N* for $z_{j}=\left(j^{2}\pm \frac{1}{2}j\right)d$. We also replace $\frac{1}{2}\gamma$ with γ in $M_{0}$. In this case, the electronic structure is periodic in *k* with period 2π and exhibits a clear hierarchy of minibands and gaps, as seen in Figure 8. Similar to the case of n=2 treated above, we see the development of a clear hierarchy of minibands and gaps—far clearer than for any GC with n>2 that we have studied and, indeed, clearer than for n=2 itself. Here, we see the trivially delocalized states at k=2mπ, the factor of 2 due to the $\frac{1}{2}j$ in $z_{j}$. Noting that the range of *k* on the horizontal axis is 2π, in contrast to Figure 6, we actually see similar, but not identical, structures within a range of π in the two figures. The connection between these two cases is commented upon later.

In fact, it would appear that $z_{j}=j^{2}d$ and $z_{j}=\left(j^{2}\pm \frac{1}{2}j\right)d$ are indeed special cases, as it would appear that only for these can we have a *j*-independent inflation rule similar to Equations (8) and (9). As we now show, these two GCs have transfer matrices sharing the same underlying structure, and thus, they have the same hidden translational invariance. To proceed, for $z_{j}=\left(j^{2}+\frac{1}{2}j\right)d$, however, instead of rule (8) and (9), we develop new rules, as shown below, that, as we shall see, are closely related. Note that the transfer matrix in this case for the *N*th generation GC is

(25) $$T_{2N+1}=Q_{0}^{(2N+1)/2}MQ_{0}^{2N/2}M\ldots Q_{0}^{6/2}MQ_{0}^{5/2}MQ_{0}^{4/2}MQ_{0}^{3/2}MQ_{0}^{2/2}MQ_{0}^{1/2}M_{0}.$$

Now, define the inflation rules

(26) $$\begin{array}{ccc} \;M & \to & Q_{0}^{1/2}MQ_{0}^{1/2}, \end{array}$$

(27) $$\begin{array}{ccc} M_{0} & \to & Q_{0}^{1/2}MQ_{0}MQ_{0}^{1/2}M_{0}Q_{0}^{1/2}. \end{array}$$

The application of these rules to $T_{2N+1}$ gives

(28) $$\begin{array}{ccc} T_{2N+3} & = & Q_{0}^{(2N+3)/2}MQ_{0}^{2N+1/2}M\ldots Q_{0}^{6/2}MQ_{0}^{5/2}MQ_{0}^{4/2}MQ_{0}^{3/2}MQ_{0}^{2/2}MQ_{0}^{1/2}M_{0}\\ & \equiv & Q_{0}^{(2N+2)/2}MQ_{0}^{2N+1/2}M\ldots Q_{0}^{6/2}MQ_{0}^{5/2}MQ_{0}^{4/2}MQ_{0}^{3/2}MQ_{0}^{2/2}MQ_{0}^{1/2}M_{0}Q_{0}^{1/2}. \end{array}$$

Note that the transfer matrix in Equation (25) for this GC has the same symmetry as the GC for n=2. Make the replacements d→4d, $M_{0}\to Q_{0}^{1/2}M_{0}Q_{0}^{1/2}$, and $M\to Q_{0}^{1/2}MQ_{0}^{1/2}$ in Equation (25) and recover a transfer matrix with the structure of that for the n=2 GC in Equation (7). Note that, however, the actual *k*-dependence of the transfer matrices in these two cases are different.

## 5. Conclusions

In this work, we have investigated the evolution of a hierarchy of minibands and gaps with increasing *N* in GCs. The work is empirical in the sense that we present numerical calculations without being able to rigorously identify the underlying physics in all cases. Hopefully, future work will provide some insight into the underlying physics. Nonetheless, based on what is presented here, we can make a number of statements based on our results.

We numerically compute the delocalized electronic states as a function of chain length *N* to see the development of a hierarchy of minibands and gaps. The cases with the most visually evident structure appear to be for the GC and the generalized quadratic GC for n=2. We observe the formation of minibands and gaps that appear to persist for high values of *N*; as *N* increases, most states appear to become localized, though some states continue to be delocalized. The integrated density of states shows a region of enhanced value near kd=π for the GC with n=2, as well as signs of self-similarity. Based on rigorous arguments developed elsewhere [14,15], perspective delocalized states have wavevectors between Gauss sites $k=k_{r,s}=\frac{r}{s}\frac{\pi }{d}$, where *r* and *s* are coprime integers. The spectrum of delocalized states is singular continuous in the N→∞ limit [14], consistent with what is noted here. This is a consequence of the *k*-dependent translational invariance of the transfer matrix.

In contrast, for n>2 (only results for n=3 are presented here), the structure seen in the evolution of the minibands and gaps with increasing *N* is visually not evident compared with that for n=2. We find that the transfer matrix exhibits a *k*-dependent translational invariance for all GCs; however, the origin of the difference between the n=2 GC and the generalized quadratic GC studied here and GCs with n>2 can be hinted at by a RSRG technique whereby the transfer matrix for an (N+1)-site GC is generated from the transfer matrix for an *N*-site GC. The iterative, bottom-up RSRG approach for computing the half-trace $x_{N}$ of the transfer matrix $T_{N}$ is presented for an *N*-site quadratic GC n=2 and for the specific generalized quadratic GC $z_{j}=\left(j^{2}\pm \frac{1}{2}j\right)d$. The approach, which does not work for n>2 or any other case, reveals a self-similar structure to the transfer matrix for the n=2 GC, as well as for the generalized quadratic GC.

## Data Availability

Data are contained within the article.
